# Threat of the Spores

**DOI:** 10.3201/eid1004.031030

**Published:** 2004-04

**Authors:** Setu K. Vora

It is difficult
to get the news from poems
yet men die miserably every day
for lack
of what is found there.
“Asphodel, That Greeny Flower” by William Carlos Williams, a physician and poet
Beware the threat of the spores,
Bad news parcels bide their time.
They lurk to enter the unsuspecting
And unleash dormant evil that multiplies.
The twin rings of code within are culprits
Spawning the Unholy Trinity of toxins.
Inject to bleed, swell, and destroy,
While the “protective” antigen does anything but.
Skin lesions black as the namesake coal,
Pale in comparison to the inhaled disease.
Finely milled, known simply as Ames strain
Artificial and abhorrent agents of terror.
“Alpha” bacteria,
Germs of the germ theory.
Origin of the famous postulates,
Crafty bugs made cruel by man.
Brought home the nesting-doll syndrome,
Envelopes, spores, bacilli, plasmids.
With it the fear and the frenzy
To cure what we create.

